# Prevalence Rates, Perceptions of Risk, and Motivations for Nonmedical Cannabis Use in Pediatric Pain

**DOI:** 10.1001/jamanetworkopen.2025.12870

**Published:** 2025-05-29

**Authors:** Joe Kossowsky, Christine Greco, Bridget A. Nestor, Camila Koike, Nicole Tacugue, Andreas M. Baumer, Elissa R. Weitzman

**Affiliations:** 1 Department of Anesthesiology, Critical Care, and Pain Medicine, Boston Children’s Hospital, Boston, Massachusetts; 2 Department of Anesthesia, Harvard Medical School, Boston, Massachusetts; 3 Division of Sleep Medicine, Harvard Medical School, Boston, Massachusetts; 4 Department of Psychology, Endicott College, Beverly, Massachusetts; 5 Institute for Implementation Science in Health Care, University of Zürich, Zürich, Switzerland; 6 Division of Addiction Medicine, Boston Children’s Hospital, Boston, Massachusetts; 7 Division of Adolescent and Young Adult Medicine, Boston Children’s Hospital, Boston, Massachusetts; 8 Department of Pediatrics, Harvard Medical School, Boston, Massachusetts; 9 Computational Health Informatics Program, Boston Children’s Hospital, Boston, Massachusetts

## Abstract

**Importance:**

Characterizing cannabis use (CU) rates in pediatric pain is critical as adolescence is a period of increased substance use and risk for negative outcomes. Youths with chronic pain may engage in CU to cope with symptoms.

**Objective:**

To examine CU rates, risk perceptions, and motivations for use among treatment-seeking youths with diagnosed pain disorders.

**Design, Setting, and Participants:**

Cross-sectional survey study conducted from September 2021 to May 2024 at a pain treatment clinic at a pediatric hospital in the Northeast United States. Participants were 251 adolescents receiving treatment for diagnosed pain conditions. Of 312 approached, 40 did not complete the survey; 17 were excluded.

**Exposures:**

Validated self-report measures assessing demographics, pain, and substance use.

**Main Outcomes and Measures:**

CU rates, motivations for use, and perceived risks.

**Results:**

Of 245 adolescents providing complete data (mean [SD] age 16.9 [1.4] years; 168 [68.6%] female; 1 [0.4%] American Indian, 3 [1.2%] Asian, 3 [1.2%] Black, 19 [7.8%] Hispanic or Latino, and 201 [82.1%] White), 62 (25.3%) endorsed lifetime CU, with a mean (SD) age at first use of 15.3 (1.9) years. Among those endorsing CU, past-year prevalence was 90.2% (56 of 62 participants) and past-month prevalence was 64.5% (40 of 62 participants). The CU group was older (mean difference, 0.9 years; 95% CI, 0.5 to 1.2 years; *P* < .001), included fewer female participants (difference, −14.0%; 95% CI, −32.6% to −2.9%; *P* = .03), and reported greater pain interference scores (mean difference, 2.7; 95% CI, 0.8-4.6; *P* = .01) and depressive symptoms scores (mean difference, 6.2; 95% CI, 2.8-9.5; *P* < .001) compared with the no CU group. The no CU group perceived cannabis as riskier (odds ratio, 2.37; 95% CI, 1.28-4.39; *P* = .01). Among youths endorsing CU, 48 of 62 (77.4%) endorsed instrumental use (IU) to alleviate psychological or physical symptoms (primarily pain, sleep, and anxiety), representing an overall IU prevalence of 19.6% (48 of 245 participants). Youths reporting IU were younger (mean difference, −0.7 years; 95% CI, −1.3 to −0.1 years; *P* = .04) and reported greater functional disability scores (mean difference, −8.4; 95% CI, 2.1 to 12.6; *P* = .01) compared with the no IU group.

**Conclusions and Relevance:**

In this cross-sectional study, approximately 25% of treatment-seeking youths with chronic pain reported CU, with more than 75% using cannabis instrumentally to treat symptoms despite limited evidence supporting cannabis for pain, sleep, or anxiety. Given CU rates in this population, education about the risks of self-medication and the development of alternative coping strategies are needed.

## Key Points

**Question:**

What are the rates of cannabis use among treatment-seeking youths with pain disorders, and how often is use associated with attempts to alleviate symptoms?

**Findings:**

In this cross-sectional study of 245 adolescents with chronic pain, 25.3% reported lifetime cannabis use, and 77.4% of users endorsed instrumental use, primarily for pain, sleep, and anxiety. Youths endorsing instrumental use had greater functional disability and more pain days than recreational users.

**Meaning:**

Despite limited evidence supporting cannabis for pain, sleep, or anxiety, these findings suggest many youths with pain use it instrumentally, underscoring the need for education on self-medication risks and development of alternative coping strategies.

## Teaser

This cross-sectional study evaluates cannabis use rates, risk perceptions, and motivations for use among treatment-seeking youths with diagnosed pain disorders.

## Introduction

Pediatric pain affects nearly 40% of youths globally^1,2,3^ and is associated with functional impairment,^4^ comorbid internalizing disorders,^5^ interpersonal stressors,^6^ and other lifestyle disruptions, including sleep disturbance.^7,8^ Among adolescents without pain disorders, these challenges often correlate with increased cannabis use (CU),^9,10,11,12^ the second most common psychotropic substance among US adolescents.^13^ In pediatric pain samples, CU prevalence is less established.

In adults with pain, CU rates are high.^14,15,16,17^ The main phytocannabinoids found in cannabis are delta 9-tetrahydrocannabinol (THC) and cannabidiol (CBD).^18^ THC is the primary psychoactive compound and is linked to potential adverse effects, including anxiety, addiction, and psychosis. In contrast, CBD is nonpsychoactive and has a relatively high toxicity threshold.^18^ Adults with CU or other substance use disorders are more likely to report pain than those without such disorders.^19^ Theoretical models explaining the high co-occurrence of pain and substance use suggest pathways of bidirectional reinforcement, whereby pain and substance use each reciprocally increase longitudinal risk for and exacerbation of the other.^20^ Adults with chronic pain often report CU to relieve pain,^21,22^ yet such instrumental use (IU) may worsen pain and substance use over time.^20^ In adults, meta-analytical findings indicate that cannabis has minimal effects on pain control,^23,24^ no significant impact on sleep,^24^ and may exacerbate anxiety^25^ compared with placebo.

Prior work shows that CU is more likely in youths with chronic medical conditions as compared with healthy peers.^26^ Many of these youths also report cannabis IU to manage pain and other discomfort.^27,28^ Studies in nationally representative youth samples have found that increased pain is associated with increases in substance use.^29,30,31^ To the authors’ knowledge, though, no study to date has investigated CU and IU rates in treatment-seeking youths with chronic pain.

Characterizing CU rates in pediatric pain is critical, as adolescence marks a developmental window of increased substance use risk^32^ and negative outcomes,^33^ like impaired cognitive function,^34^ increased suicide risk,^35,36^ and poor sleep.^35^ Recent work indicates age-dependent associations between CU and psychosis, particularly during adolescence, highlighting the vulnerability of this development stage.^37,38^ Understanding why youths with pain engage in CU or IU can guide interventions toward more adaptive coping skills. The current survey study aims to examine CU rates, risk perceptions, and motivations for use or abstinence in a treatment-seeking sample of youths with diagnosed pain disorders. We also explored differences in these variables across different subgroups (eg, youth with CU vs no CU and youths with IU vs no IU). We hypothesized that youths who report IU perceive cannabis as less harmful than youths with no CU and no IU, which, to some extent, may explain their increased CU.

## Methods

### Participants and Procedures

Participants were recruited from a pediatric pain clinic in the Northeast US, in a state where both medical and recreational cannabis use are legal for adults aged 21 years and older. This study occurred from September 2021 to May 2024. A sample size of 200 was calculated to detect small-to-moderate differences (d = 0.4, α = 0.05, power = 0.8) between cannabis users (estimated 25% prevalence) and nonusers. To account for potential nonresponses, 312 patients were approached, 251 enrolled, and 245 completed surveys. Participants aged 14 to 19 years receiving treatment for diagnosed chronic pain conditions (headache or migraine, whole-body pain, joint pain, or neuropathic pain) were eligible. Exclusion criteria included severe cognitive impairment or inability to respond to questions in English. Eligible patients were identified weekly from clinic schedules and recruited via phone, email, mail, secure patient portal, or in-person. Participants and their guardians provided informed consent or assent. Surveys were administered securely online via REDcap.^39,40^ Participants received a $20 gift card. The study was approved by the Boston Children’s Hospital institutional review board and adheres to Strengthening the Reporting of Observational Studies in Epidemiology (STROBE) reporting guidelines.

### Measures

#### Demographics

Demographic questions assessed participant’s age, grade, race (American Indian, Asian, Black, White, and other or multiple races), ethnicity (Hispanic or Latino, not Hispanic or Latino, or prefer not to answer), and self-reported gender. Race and ethnicity were assessed in this study because of previously documented differences in youth cannabis use in different racial and ethnic groups.^41^

#### Pain

Participants completed the Numerical Rating Scale^42^ to assess mean pain intensity on a 0 to 10 scale (0 = no pain to 10 = worst pain imaginable), and the Pain Frequency and Duration Scale^43^ to characterize pain symptoms experienced over the past 2 weeks. Participants also completed the 8-item Patient Reported Outcomes Measurement Information System Pain Interference scale,^44^ which measures pain-related disruptions across multiple life domains, and has demonstrated good reliability and validity in youths^45^ and adults.^46^ Participants indicate the degree to which pain hinders both physical and psychosocial functioning in certain activities (1, never; 2, almost never; 3, sometimes; 4, often; or 5, almost always). Participants completed pediatric (<18 years) or adult (≥18 years) versions of this measure. Summed raw scores were converted into *t* scores, with higher scores indicating greater pain interference. Reliability was excellent (Cronbach α= 0.86 pediatric; 0.95 adult).

Participants also completed the 15-item Functional Disability Inventory,^47^ which assesses perceived difficulty completing physical activities in school, home, or recreational settings (0, no trouble; 1, a little trouble; 2, some trouble; 3, a lot of trouble; or 4, impossible). Raw scores were summed to a total score (0-60). Higher scores indicate greater functional pain disability. Cronbach α was 0.91, suggesting excellent reliability.

#### Cannabis Use

Participants were asked whether they had used cannabis at any point in their lifetime, in the past year, and in the past month, and to specify their age at first use. Those who endorsed CU then completed items regarding frequency, motives, perceived risks, beliefs, and likelihood of future use. Items were selected and combined from several validated self-report questionnaires.^48,49^ Further information is in eAppendix 1 in Supplement 1.

##### Frequency

Participants estimated the number of CU occasions (0 to ≥40) over their lifetime, past year, past month, and on a typical day. Participants with CU specified their most frequent methods of cannabis ingestion (eg, joints, hookah, edibles, and so forth). See eAppendix 2 in Supplement 1 for a sample question.

##### Motivations

Participants with CU reported (yes vs no) whether alleviating physical or psychological symptoms motivated their use (ie, IU). If endorsed, participants identified such symptoms (eg, pain, nausea, anxiety, and so forth). Participants endorsed (yes vs no) whether their CU was upon a physician’s recommendation (ie, medical marijuana). Participants also reported how frequently they used cannabis instead of alcohol for pain (1, never; 2, rarely; 3, sometimes; 4, often; or 5, all the time).

##### Adverse Effects, Importance of Risks, and Likelihood of Future Use

Participants with CU reported past-year frequency of hallucinations, anxiety, or paranoia during or after CU (1, never; 2, once or twice; 3, sometimes; 4, often). Participants without CU indicated the extent to which CU risks were important to them (1, not at all; 2, somewhat; or 3, very important) including psychological or physical damage, addiction, and financial expense. Participants with CU reported their estimated likelihood of future CU in the next 30 days and the next year (1, definitely will; 2, probably will; 3, probably will not; or 4, definitely will not).

##### Beliefs

All participants reported their and their parents’ beliefs regarding riskiness of regular CU (1, risk; 2, slight risk; 3, moderate risk; 4, great risk; or 5, unsure risk). All participants also reported the extent to which they agreed or disagreed (1, strongly disagree; 2, disagree; 3, neither agree nor disagree; 4, agree; or 5, strongly agree) with commonly held cannabis beliefs (eg, “Cannabis can be addictive”).

### Statistical Analysis

Python version 3.8.20 (Python Software Foundation) was used for descriptive analyses of demographic, pain, and CU variables and for inferential statistics. Independent *t* tests compared age, pain intensity, number of pain days, functional disability, and pain interference variables between participants with CU vs no CU, and between those with IU vs no IU. Monte Carlo χ^2^ tests compared gender, race, ethnicity, and pain characteristics across groups. Beliefs response categories were grouped with “strongly agree” and “agree” combined and “neither agree nor disagree,” “disagree,” and “strongly disagree” combined. Risk perception responses were categorized into low risk (“no risk” and “slight risk”) and higher risk (“moderate risk” and “great risk”). χ^2^ tests were conducted for these categorical comparisons. Pairwise deletion was applied for missing data, which accounted for 1.59% of responses. A 2-sided *P* value less than .05 was considered significant.

## Results

The total sample consisted of 251 adolescents (mean [SD] age 16.9 [1.4] years; 168 [68.6%] female; 1 [0.4%] American Indian, 3 [1.2%] Asian, 3 [1.2%] Black, 19 [7.8%] Hispanic or Latino, and 201 [82.1%] White) with diagnosed pain disorders; 245 (97.6%) provided complete data and were included in the final analysis. Of 312 eligible patients approached, 40 did not complete the survey, and 4 were excluded from the analysis due to being either out of study age range, scam person, or a test participant. Our sample did not differ significantly in age or gender from the pediatric chronic pain population seen at the hospital where data were collected (Table 1).

**Table 1.** Participant’s Cannabis Use (CU) Characteristics^a^

Characteristic	Participants, No. (%)			*P *value^b^	Participants, No. (%)		*P *value^c^
	Total sample (N = 245)	No CU (n = 183)	Total lifetime CU (n = 183)		Within CU group		
					IU (n = 48)	No IU (n = 14)	
Demographics							
Age, mean (SD), y	16.91 (1.42)	16.7 (1.4)	17.6 (1.2)	<.001^d^	17.4 (1.2)	18.1 (1)	.04^d^
Self-reported gender							
Female	168 (68.6)	132 (72.1)	36 (58.1)	.03^d^	23 (47.9)	13 (92.9)	.05
Male	36 (14.7)	24 (13.1)	12 (19.4)		11 (22.9)	1 (7.1)	
Nonbinary	25 (10.2)	13 (7.1)	12 (19.4)		12 (25.0)	0	
Prefer not to answer	7 (2.8)	6 (3.3)	1 (1.6)		1 (2.1)	0	
Prefer to self-describe	9 (3.7)	8 (4.4)	1 (1.6)		1 (2.1)	0	
Ethnicity							
Hispanic or Latino	19 (7.8)	13 (7.1)	6 (9.7)	.57	5 (10.4)	1 (6.2)	.80
Not Hispanic or Latino	224 (91.4)	169 (92.3)	55 (88.7)		13 (92.9)	42 (87.5)	
Prefer not to answer	1 (0.8)	1 (0.5)	1 (1.6)		1 (2.1)	0	
Race							
American Indian	1 (0.4)	0	1 (1.6)	.12	0	1 (7.1)	.05
Asian	3 (1.2)	1 (0.6)	2 (3.2)		2 (4.2)	0	
Black	3 (1.2)	3 (1.6)	0		0	0	
White	201 (82.1)	153 (83.6)	48 (77.4)		35 (72.9)	13 (92.9)	
Other or multiple races	37 (15.1)	26 (14.2)	11 (17.8)		11 (22.9)	0	
Pain characteristics, mean (SD)							
Pain days in the last 2 wk	11.8 (3.7)	11.8 (3.7)	11.9 (3.3)	.76	12.0 (2.7)	10.4 (4.6)	.14
Usual pain intensity	5.5 (1.7)	5.6 (1.8)	5.3 (1.5)	.16	5.3 (1.5)	5.4 (1.4)	.65
Worst pain intensity	7.8 (1.7)	7.9 (1.7)	7.7 (1.7)	.46	7.7 (1.5)	7.1 (2.2)	.20
Functional disability	23.9 (11.5)	23.4 (12.1)	25.5 (9.6)	.18	27.4 (8.7)	19.0 (10.2)	.01^d^
Pain interference pediatric (n = 177)^e^	60.0 (7.3)	59.6 (7.7)	62.3 (4.5)	.01^d^	61.8 (4.8)	61.8 (2.5)	.67
Pain interference adults (n = 66)^e^	60.0 (8.1)	59.2 (8.3)	61.8 (7)	.12	62.6 (4.2)	58.9 (10.3)	.25
Pain regularity							
Recurrent	66 (26.3)	50 (27.3)	16 (25.8)	.74	11 (22.9)	5 (35.7)	.33
Continuous	180 (71.7)	129 (70.5)	46 (74.2)		37 (77.1)	9 (64.3)	
Psychological characteristics, *t* score, mean (SD)							
Depressive symptoms pediatric	56.9 (11.6)	55.6 (12.0)	61.8 (8.3)	<.001^d^	63.1 (7.4)	54.9 (10.6)	.12
Depressive symptoms adults	55.6 (9.2)	54.9 (9.7)	56.6 (8.4)	.46	58.8 (7.4)	52.1 (8.9)	.07
Anxiety pediatric	56.5 (11.5)	56.0 (11.7)	58.5 (10.5)	.22	58.5 (10.9)	58.5 (9.0)	>.99
Anxiety adult	57.4 (10.3)	56.0 (11.6)	59.4 (7.9)	.15	61.4 (7.0)	55.6 (8.6)	.10

Abbreviation: IU, instrumental use.

^a^ Comparisons between CU and no CU groups reflect differences within the full sample. Comparisons between IU and no IU groups reflect differences within cannabis users. Percentages in IU and no IU columns are based on lifetime cannabis users (62 participants), not the full sample. Overall IU prevalence in the full sample is 19.6% (48 of 245 participants).

^b^ *P* values represent differences between CU and No CU groups.

^c^ *P* values represent differences between IU and No IU groups.

^d^ Indicates significance.

^e^ Indicates *t* scores.

### Group Differences of Participant Characteristics

Participants with CU were older (mean difference, 0.9 years; 95% CI, 0.5 to 1.2 years; *P* < .001) and included fewer female participants (difference, −14.0%; 95% CI, −32.6% to −2.9%; *P* = .03) compared with those without CU. The pediatric CU group reported higher pain interference scores (mean difference, 2.7; 95% CI, 0.8 to 4.6; *P* = .01) and higher depressive symptoms scores (mean difference, 6.2; 95% CI, 2.8 to 9.5; *P* < .001) than the no CU group. Participants with IU were younger (mean difference, −0.7 years; 95% CI, −1.3 to −0.1 years; *P* = .04) and reported significantly higher functional disability scores (mean difference, 8.4; 95% CI, 2.1 to 12.6; *P* = .01**)** compared with those with no IU. No other significant differences emerged between CU vs no CU or IU vs no IU groups (Table 1**)**.

### Cannabis Use (CU)

Participant lifetime CU was 62 of 245 participants (25.3%), and age of first CU ranged from 7 to 19 years (mean [SD] age, 15.3 [1.9] years). Prevalence rates for past year and past month were 22.9% (56 of 245 participants) and 16.3% (40 of 245 participants), respectively. Among those endorsing CU, past-year prevalence was 90.2% (56 of 62 participants) and past-month prevalence was 64.5% (40 of 62 participants).

#### Frequency

Frequency of CU ranged from 0 to more than 40 occasions. For lifetime use, median use was 6 to 9 occasions, with most participants endorsing use on either 3 to 5 occasions (15 of 62 participants [24.2%]) or on more than 40 occasions (16 of 62 participants [25.8%]). Median use over the past year was 6 to 9 occasions, and most participants endorsed use on 3 to 5 occasions (15 of 62 participants [24.2%]). Over the past month, median use was 0 occasions, and most participants reported either 0 (22 of 62 participants [35.5%]) or 1 to 2 occasions of use (19 of 62 participants [30.6%]). On a typical day of cannabis use, 56 of 62 participants (90.3%) reported using 1 to 2 times.

Preferred methods for ingesting cannabis were edibles (32 of 62 participants [51.6%]), vaporizer (28 of 62 participants [45.2%]), joints (27 of 62 participants [43.5%]), bong (15 of 62 participants [24.2%]), blunts (8 of 62 participants [12.9%]), and hand pipe (8 of 62 participants [12.9%]) (see eTable in Supplement 1 for additional information). The primary form of CU varied, with 37 of 62 participants (59.7%) endorsing marijuana with high THC content, 24 of 62 (38.7%) endorsing edibles, 20 of 62 (32.3%) endorsing marijuana with high CBD content, 9 of 62 (14.5%) endorsing concentrates, and 2 of 62 (3.2%) endorsing other unspecified methods.

#### Adverse Experiences

Participants reported experiencing hallucinations never (51 of 62 participants [82.3%]), once or twice (8 of 62 participants [12.9%]), sometimes (1 of 62 participants [1.6%]), or often (2 of 62 participants [3.2%]). They reported anxiety or paranoia never (47 of 62 participants [75.8%]), once or twice (11 of 62 participants [17.7%]), sometimes (3 of 62 participants [4.8%]), or often (1 of 62 participants [1.6%]) during or after CU over the past year.

#### Motivations for Use and Nonuse

Rates of CU to alleviate physical or psychological symptoms were 77.4% (48 of 62 participants), with 93.8% (45 of 48 participants) endorsing use for pain, 72.9% (35 of 48 participants) for sleep, 68.8% (33 of 48 participants) for anxiety, 33.3% (16 of 48 participants) for nausea, 31.3% (15 of 48 participants) for appetite, and 6.3% (3 of 48 participants) for other unspecified symptoms. The overall IU prevalence was 19.6% (48 of 245 participants) in the full sample, highlighting that nearly 1 in 5 youths with pain reported CU specifically for symptom relief (Figure).

**Figure.**
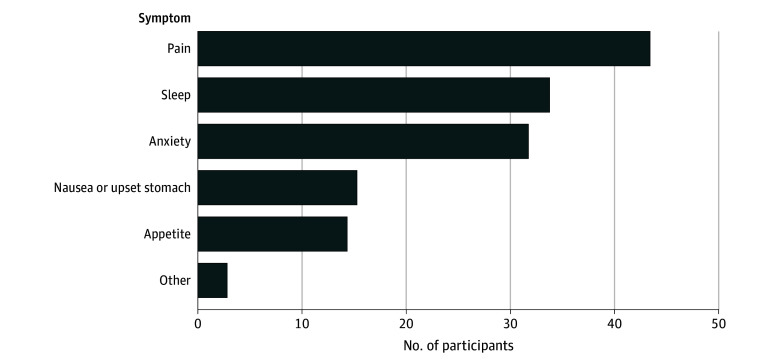
Primary Symptoms Targeted by Instrumental Cannabis Use Among Adolescents With Chronic Pain Participants responded to the question, “For which of the following symptoms or side effects did you use cannabis?”

Also, 7 of 62 participants (11.3%) endorsed current medical CU, indicating their CU was upon a physician’s recommendation, and 1 of 62 (1.6%) endorsed medical CU in the past. Participants indicated how often they chose cannabis over alcohol because they perceived alcohol to worsen their pain: 34 of 62 participants (54.8%) chose never, 5 of 62 (8.1%) chose rarely, 4 of 62 (6.5%) chose sometimes, 8 of 62 (12.9%) chose often, and 11 of 62 (17.7%) chose all the time.

Participants with no CU reported the extent to which cannabis-associated risks were important to them (Table 2). More than 50% reported the following possible negative consequences to be very important to them: becoming addicted (117 of 183 participants [63.9%]), physical damage (109 of 183 participants [59.6%]), leading to stronger drugs (103 of 183 participants [56.3%]), don’t feel like getting high (102 of 183 participants [55.7%]), losing control (101 of 183 participants [55.2%]), psychological damage (100 of 183 participants [54.6%]), and parents would disapprove (92 of 183 participants [50.3%]).

**Table 2.** Stated Reasons for Not Using Cannabis (N = 183)

Reason	Participants, No. (%)		
	Not at all important	Somewhat important	Very important
Concerned about psychological damage	33 (18)	50 (27.3)	100 (54.6)
Concerned about physical damage	34 (18.6)	40 (21.9)	109 (59.6)
Concerned about getting arrested	47 (25.7)	55 (30.1)	81 (44.3)
Concerned about becoming addicted	27 (14.8)	39 (21.3)	117 (63.9)
It’s against my beliefs	98 (53.6)	33 (18)	52 (28.4)
Concerned about loss of energy or ambition	51 (27.9)	62 (33.9)	70 (38.3)
Concerned about possible loss of control of myself	37 (20.2)	45 (24.6)	101 (55.2)
It might lead to stronger drugs	41 (22.4)	39 (21.3)	103 (56.3)
Not enjoyable, I wouldn’t like it	51 (27.9)	50 (27.3)	82 (44.8)
My parents would disapprove	45 (24.6)	46 (25.1)	92 (50.3)
My boyfriend/girlfriend would disapprove	116 (63.4)	18 (9.8)	49 (26.8)
I wouldn’t like being with the people who use it	72 (39.3)	41 (22.4)	70 (38.3)
My friends don’t use it	79 (43.2)	48 (26.2)	56 (30.6)
Too expensive	94 (51.4)	43 (23.5)	46 (25.1)
Not available	117 (63.9)	36 (19.7)	30 (16.4)
Don’t feel like getting high	35 (19.1)	46 (25.1)	102 (55.7)

#### Cannabis-Related Beliefs and Risk-Perception

Results of χ^2^ tests indicated participants with CU endorsed more agreement with the belief that “cannabis is safe because it is natural” (odds ratio [OR], 2.76; 95% CI, 1.48-5.12: *P* = .001) compared with participants with no CU. Participants with no CU indicated more agreement with the belief that “cannabis can be addictive” (OR, 0.28; 95% CI, 0.13-0.64; *P* = .003) and “using cannabis can make my medical condition worse” (OR, 0.17; 95% CI, 0.09-0.33; *P* < .001). No other significant differences emerged in cannabis-related beliefs between the IU and no IU groups. (see Table 3). Overall, most participants reported they believed regular CU would carry slight (64 of 244 participants [26.2%]) or moderate (64 of 244 participants [26.2%]) risk. Most participants further reported their parent or guardian would believe that regular CU would harm their (ie, participants’) health (162 of 244 participants [66.4%]) vs help their health (36 of 244 participants [14.8%]) or have no impact (46 of 244 participants [18.9%]). Similarly, χ^2^ tests showed that participants with no CU reported higher perceived risk of CU compared with those with CU (OR, 2.37; 95% CI, 1.28-4.39; *P* = .01). No other significant differences in perceived CU risks between the IU and no IU groups.

**Table 3.** Beliefs About Cannabis Use (CU)

Belief	Participants, No. (%)			*P *value^a^	Participants, No. (%)		*P *value^b^
	Total (N = 245)	No CU (n = 183)	Lifetime-CU (n = 62)		No IU (n = 14)	IU (n = 48)	
Cannabis is safe because it is natural							
Strongly agree/agree	130 (53.1)	86 (47)	44 (71)	<.001	8 (57.1)	36 (75)	.34
Neither agree nor disagree/disagree/strongly disagree	115 (46.9)	97 (53)	18 (29)		6 (42.9)	12 (25)	
Cannabis can be addictive							
Strongly agree/agree	217 (88.6)	169 (92.3)	48 (77.4)	.003	12 (85.7)	36 (75)	.63
Neither agree nor disagree/disagree/strongly disagree	28 (11.4)	14 (7.7)	14 (22.6)		2 (14.3)	12 (25)	
Using cannabis can make my medical condition worse							
Strongly agree/agree	182 (74.3)	153 (83.6)	29 (46.8)	<.001	10 (71.4)	19 (39.6)	.07
Neither agree nor disagree/disagree/strongly disagree	63 (25.7)	30 (16.4)	33 (53.2)		4 (28.6)	29 (60.4)	
Risk of harm associated with regular cannabis use (n = 244)							
Low risk: risk/slight risk	108 (44.3)	82 (45.0)	26 (41.9)	.009	8 (57.1)	18 (37.5)	.32
High risk: moderate risk/great risk	84 (34.4)	48 (26.4)	36 (58.1)		6 (42.9)	30 (63.5)	
Can’t say, not familiar with the drug	52 (21.3)	52 (28.6)	0	NA	0	0	NA

Abbreviations: IU, instrumental use; NA, not applicable.

^a^ *P* values relate to differences between total CU and no CU.

^b^ *P* values relate to differences between IU and no IU.

#### Likelihood of Future Use

For those with CU, when asked about the likelihood of CU in the next month, 16 of 62 participants (25.8%) reported they definitely will, 16 of 62 (25.8%) probably will, 17 of 62 (27.4%) probably will not, and 13 of 62 (21.0%) definitely will not. When asked about likelihood of CU in the next year, 24 of 62 (38.7%) reported they definitely will, 25 of 62 (40.3%) probably will, 7 of 62 (11.3%) probably will not, and 6 of 62 (9.7%) definitely will not.

## Discussion

This study examined CU and IU rates in youths with diagnosed pain disorders. Lifetime CU prevalence was approximately 25%, with past year and past month rates of 23% and 16%, respectively, which is similar to those of nationally-representative adolescent samples (lifetime: 22.4%, past year: 17.8%, past month: 10.3%).^50^ The mean age of first use was 15 years, raising concerns, since initiating CU at younger ages (12-17 years) doubles the risk of developing CU disorders compared with initiating later.^51^ Additionally, youths with CU demonstrated a bimodal distribution, typically using cannabis either occasionally (3-5 occasions) or heavily (over 40 occasions). Taken together, these results highlight unique vulnerability for CU among youths with diagnosed pain disorders.^52^ Early identification of high-risk groups remains crucial.

Youths with CU reported greater pain interference than youths with no CU. One explanation is that more severe pain increases CU, potentially exacerbating pain interference. Alternatively, both cannabis use and pain interference may share an underlying driver such as depressive symptoms, which could increase both pain sensitivity and substance use likelihood. Further, CU influences endogenous pain systems through activation of cannabinoid receptor 1,^53^ setting youths up for a vicious cycle of increased pain and urge of use.^54^ Nearly 80% of youths reporting CU endorsed IU primarily for pain, anxiety, and sleep disturbances. This IU prevalence is higher than reported among other pediatric populations with chronic conditions^27^ but similar to adult populations using cannabis for pain, stress, and sleep relief.^55,56^ The high IU prevalence suggests that CU among youths with pain may be influenced by a complex interplay of biological (pain severity),^53^ psychological (anxiety or sleep disturbances), and social (peer influence or accessibility) factors, highlighting the importance of multidimensional interventions beyond pain management alone. Youths reporting IU also had higher functional disability than those with no IU, possibly reflecting unsuccessful attempts at using more adaptive coping strategies or elevated symptom severity, leaving CU for symptom relief as their only effective strategy. Future work should explore contextual factors associated with IU to enhance targeted interventions.

Findings regarding cannabis risk perceptions revealed youths with CU perceived cannabis as less harmful than their peers without CU. This discrepancy may result from greater exposure among those with use to cannabis’ purported analgesic properties, shaping their perceptions of risk and benefits. Given mixed evidence of cannabis’ effectiveness for pain relief,^24,25^ this underscores the importance of educating youths about cannabis risks in clinical settings, particularly for those who respond poorly to conventional treatments. Integrating adaptive coping skills training may reduce reliance on cannabis, potentially improving treatment outcomes.

Finally, our findings also reveal reasons why some youths with pain may not use cannabis. Most youths in our sample with no CU reported concerns about psychological or physical damage, addiction, and loss of control as very important to them. Most of these youths also reported they did not feel like getting high and that their parents would disapprove if they were to use cannabis. This information may help clarify what beliefs distinguish youths with pain who do vs do not use cannabis and may ultimately guide supportive strategies for abstinence.

### Limitations

Limitations of this study also provide avenues for future research. First, this study is cross-sectional, so temporal or causal claims about CU trajectories cannot be determined. Inclusion of a control group along with a longitudinal design would be necessary for determining true associations and clarifying fluctuations in pain and CU over time. Second, the current sample was drawn from 1 pediatric hospital in the northeast where CU is legal, which may influence youths’ perceptions of cannabis-related risks and beliefs.^57,58^ Although their demographics largely mirror those of other larger samples of pediatric pain patients,^59^ future work should seek more diverse samples to investigate CU rates across different geographic locations, as well as among patients with varying gender, sexual orientation, and cultural identities. Third, while our statistical comparisons were hypothesis-driven rather than exploratory, we acknowledge that multiple testing can increase the risk of type I error, and we report CIs and effect sizes alongside *P* values to provide a more complete interpretation of findings. Additionally, the current study lacked a measure of social desirability, which may have biased responses and did not include certain relevant demographic factors, such as socioeconomic status.

## Conclusions

In this cross-sectional study of treatment-seeking youths with pediatric pain, approximately 25% of participants reported CU, and more than 75% of cannabis-using youths reported IU for symptom relief. Given the immediate and longer-term risks associated with youth CU in pain populations,^20,60^ these findings highlight the importance of targeted education about cannabis risks and the need to develop alternative coping strategies in pediatric pain care.

## Notes

Supplement 1. **eAppendix 1. **Cannabis Use Questions **eAppendix 2.** Sample Question **eTable. **Preferred Methods for Ingesting Cannabis

Supplement 2.
Data Sharing Statement
