# Supplementary material for: Prevalence Rates, Perceptions of Risk, and Motivations for Nonmedical Cannabis Use in Pediatric Pain
Source: JAMA Netw Open. 2025 May 29;8(5):e2512870. doi: 10.1001/jamanetworkopen.2025.12870 (PMC12123470; doi:10.1001/jamanetworkopen.2025.12870)
Supplement: Supplement 1. — eAppendix 1. Cannabis Use Questions eAppendix 2. Sample Question eTable. Preferred Methods for Ingesting Cannabis [file jamanetwopen-e2512870-s001.pdf]

Supplemental Online Content

Kossowsky J, Greco C, Nestor BA, et al. Prevalence rates, perceptions of risk, and motivations for nonmedical cannabis use in pediatrics. *JAMA Netw Open*. 2025;8(5):e2512870. doi:10.1001/jamanetworkopen.2025.12870

eAppendix 1. Cannabis Use Questions
eAppendix 2. Sample Question
eTable. Preferred Methods for Ingesting Cannabis

This supplemental material has been provided by the authors to give readers additional information about their work.

© 2025 Kossowsky J et al. *JAMA Network Open*.

eAppendix 1: Cannabis Use Questions

The Substance Use Survey comprises questions from different validated surveys and new questions from our own repository. We assessed the uses, frequency, intention, and beliefs of most frequently substances used by adolescents with chronic pain: marijuana, alcohol, tobacco, vape, and opioid.

National Survey on Drug Use and Health (NSDUH) 2019: NSDUH questionnaire reports the prevalence, incidence, and patterns of illicit substance use and alcohol use among adolescents over 12 years. We adapted a question to marijuana use ("Think about the first time you first used cannabis. How old were you when you tried cannabis for the first time?").

Monitoring the Future (MTF) 2020: MTF is a long-term substance use study among U.S. adolescents, and adults until the age of 60 years. The included questions were related to the number of occasions participants used marijuana ("On how many occasions (if any) have you used marijuana (weed, pot) or hashish (hash, hash oil) in your 30 days/12 months?"). Questions were modified to accommodate the aim of this study, which is substance use among adolescents suffering from chronic pain ("On how many occasions (if any) have you used cannabis (weed, pot) or hashish (hash, hash oil) in your condition?"). Similarly, we inquired about medical marijuana use ("Have you ever used 'medical marijuana;' that is, cannabis you used because a doctor told you to use it?"). Additionally, we asked how frequently is the use of marijuana in certain places ("During the LAST 12 MONTHS, how often (if ever) have you used cannabis in each of the following places? At your home; at friends' house; At a school dance; a game, or other event; at school during the day; near school; In a car; and, at a party"). We assessed the intention of cannabis use ("How likely is it that you will use cannabis in the next 30 days?"). This question was modified to comprise 12 months' intention to use marijuana, alcohol use ("How likely is it that you will drink alcoholic beverages in the next 30 days/ 12 months?"). For adolescents who reported never using marijuana in their lifetime, we inquire about their motive ("Here are some reasons people give for not using marijuana or for stopping use. How important is each of the following as a reason for YOU not using marijuana? Concerned about possible psychological damage; Concerned about possible physical damage; Concerned about getting arrested;

© 2025 Kossowsky J et al. *JAMA Network Open*.

Concerned about becoming addicted; It's against my beliefs; Concerned about loss of energy or ambition; Concerned about possible loss of control of myself; It might lead to stronger drugs; Not enjoyable, I wouldn't like it; My parents would disapprove; My boyfriend/girlfriend would disapprove; I wouldn't like being with the people who use it; My friends don't use it; Too expensive; Not available; Don't feel like getting high"). We included a question from this survey regarding beliefs of marijuana ("How much do you think people risk harming themselves (physically or in other ways), if they use cannabis regularly?"), tobacco ("In your opinion, how addictive is smoking cigarettes regularly?"), and vape ("In your opinion, how addictive is vaping regularly?").

Repository questions: we included questions related to substance use ever in life ("Have you used cannabis (weed, pot) or hashish (hash, hash oil) in your lifetime?", "Have you ever had any alcoholic beverage to drink, more than just a few sips?", "Have you ever vaped (nicotine, marijuana, or just flavoring)?", and "Have you ever smoked tobacco/nicotine?"). If the adolescent reported marijuana use, further questions related to frequency ("On a typical day that you use cannabis, how many times do you use it?"), and characterization of the marijuana use ("Which of the following methods to ingest cannabis do you use regularly (at least 25% of the time you use cannabis)?", "Which other methods to ingest cannabis do you use regularly?", "What is the primary form of cannabis you use?", "What is the primary form of cannabis you use?", "Have you used cannabis to alleviate physical or psychological symptoms?", " physical or psychological symptoms?", "Which other symptoms or side effects did you use cannabis?", and "When you used cannabis in the past year, how often did you choose it instead of alcohol because you thought alcohol was worse for your pain?"). All participants had to agree or disagree with the following statements about beliefs of marijuana: "Cannabis is safe because it is natural", "Cannabis can be addictive", and "Using cannabis can make my medical condition worse". Further questions around beliefs of marijuana use: "most likely thinks cannabis would...", "In the past 12 months, how often have you seen, felt, or heard things that were not really there (i.e. hallucinations) during or after using/felt anxious or paranoid during or after using cannabis?".

© 2025 Kossowsky J et al. *JAMA Network Open*.

eAppendix 2: Sample question

The question that assessed frequency of cannabis use was: "On how many occasions (if any) have you used cannabis (weed, pot) or hashish (hash, hash oil) in your lifetime?". The options were: "0 occasion"; "1-2 occasions"; "3-5 occasions"; "6-9 occasions"; "10-19 occasions"; "20-39 occasions"; and "More than 40".

eTable. Preferred Methods for Ingesting Cannabis (N=62)

**Method**	**Frequency, n (%)**
Edibles	33 (51.6)
Vaporizer (e.g. vulcano, vape pen)	28 (45.2)
Joints	27 (43.5)
Bong (water pipe)	15 (24.2)
Blunts (cigar sized joints)	8 (12.9)
Hand pipe	8 (12.9)
Hookah	1 (1.6)
None	5 (8.1)
Other	1 (1.6)

Note: N: total number of participants; n: number of participants.

© 2025 Kossowsky J et al. *JAMA Network Open*.
