# Supplementary material for: Prevalence Rates, Perceptions of Risk, and Motivations for Nonmedical Cannabis Use in Pediatric Pain
Source: JAMA Netw Open. 2025 May 29;8(5):e2512870. doi: 10.1001/jamanetworkopen.2025.12870 (PMC12123470; doi:10.1001/jamanetworkopen.2025.12870)
Supplement: Supplement 2. — Data Sharing Statement [file jamanetwopen-e2512870-s002.pdf]

Data Sharing Statement

Kossowsky. Prevalence Rates, Perceptions of Risk, and Motivations for Nonmedical Cannabis Use in Pediatrics. *JAMA Netw Open*. Published May 29, 2025.
doi:10.1001/jamanetworkopen.2025.12870

Data
Data available: No

Additional Information
Explanation for why data not available: Data would be available upon reasonable request to the corresponding author.
